# The Binary Toxin of *Clostridioides difficile* Alters the Proteome and Phosphoproteome of HEp-2 Cells

**DOI:** 10.3389/fmicb.2021.725612

**Published:** 2021-09-14

**Authors:** Florian Stieglitz, Ralf Gerhard, Andreas Pich

**Affiliations:** ^1^Institute of Toxicology, Hannover Medical School, Hanover, Germany; ^2^Core Facility Proteomics, Hannover Medical School, Hanover, Germany

**Keywords:** binary toxin, *Clostridioides difficile*, proteome, phosphoproteome, signaling

## Abstract

*Clostridioides difficile* is a major cause of nosocomial infection worldwide causing antibiotic-associated diarrhea and some cases are leading to pseudomembranous colitis. The main virulence factors are toxin A and toxin B. Hypervirulent strains of *C. difficile* are linked to higher mortality rates and most of these strains produce additionally the *C. difficile* binary toxin (CDT) that possesses two subunits, CDTa and CDTb. The latter is responsible for binding and transfer of CDTa into the cytoplasm of target cells; CDTa is an ADP ribosyltransferase catalyzing the modification of actin fibers that disturbs the actin vs microtubule balance and induces microtubule-based protrusions of the cell membrane increasing the adherence of *C. difficile*. The underlying mechanisms remain elusive. Thus, we performed a screening experiment using MS-based proteomics and phosphoproteomics techniques. Epithelial Hep-2 cells were treated with CDTa and CDTb in a multiplexed study for 4 and 8 h. Phosphopeptide enrichment was performed using affinity chromatography with TiO2 and Fe-NTA; for quantification, a TMT-based approach and DDA measurements were used. More than 4,300 proteins and 5,600 phosphosites were identified and quantified at all time points. Although only moderate changes were observed on proteome level, the phosphorylation level of nearly 1,100 phosphosites responded to toxin treatment. The data suggested that CSNK2A1 might act as an effector kinase after treatment with CDT. Additionally, we confirmed ADP-ribosylation on Arg-177 of actin and the kinetic of this modification for the first time.

## Introduction

*Clostridioides difficile* infections are with 223,900 cases in hospitalized patients in 2017, a significant cause for nosocomial infections, and classified by the Centers for Disease Control and Prevention as an urgent threat with 12,800 deaths in 2017 in the United States (Centers for Disease Control and Prevention, 2019). The main virulence factors of *C. difficile* are the large glycosylating toxins TcdA and TcdB that target small GTPases of the Ras and Rho subfamily. Our group studied the effects of TcdA and TcdB on target cells concerning the glycosyltransferases effect via proteomic experiments. Both toxins alter the proteome of colonic cells in a time-dependent manner and effect proteins that are involved in cellular functions related to the cytopathic and cytotoxic effect of the large glucosylating toxins. Mutant TcdA with impaired glucosyltransferase activity showed no effect on the proteome of target cells; in contrast, glucosyltransferase inactive TcdB induces in epithelial cells (HEp2) a process called pyknosis that is accompanied by extensive alterations on the proteome level (Zeiser et al., 2011, 2013; Jochim et al., 2014; Erdmann et al., 2017; Junemann et al., 2017).

In recent years hypervirulent strains of *C. difficile* (e.g., NAP1/BI/027) emerged that produce additionally the binary toxin (CDT; Aktories et al., 2018). Up to 30% of clinical isolates that produce the binary toxin are linked to increased morbidity and mortality rates (Abeyawardhane et al., 2021). CDT belongs to the iota toxin family and consists of two separated subunits, the catalytically active CDTa and the pore-forming CDTb. CDT acts differently from TcdA and TcdB by ribosylating G-actin directly, subsequently prohibiting the prolongation of the F-actin, leading to depolymerization of F-actin and an imbalance of the actin microtubule homeostasis. While the actin cytoskeleton collapses, the cells form protrusions based on microtubule polymerization (Schwan et al., 2009). It has been advocated that the microtubule-based protrusions were beneficial for attachment of *C. difficile* to the epithelia. Septins play a significant role in this process since they are the primary driver of microtubule polymerization guidance (Schwan et al., 2009). Recent studies have shown that the knockout of septin prohibits microtubule-based protrusions and CDC42 involvement in septin regulation (Nölke et al., 2016). However, the underlying mechanism of microtubule stabilization and general cell response to CDT remains elusive. It has always been assumed that CDT ribosylates actin on Arg-177 like other iota-type toxins, but it has never been directly shown while general actin ribosylation by CDT has been proven (Vandekerckhove et al., 1987; Gülke et al., 2001).

This study investigated the fundamental mechanism in the cell response to CDT treatment of human epithelial cells (HEp-2) by LC-MS-based proteomics utilizing a tandem mass tags (TMT)-based phosphoproteomic approach. In a two-timepoint experiment, a detailed global phosphoproteomic analysis was carried out, and the catalytical active subunit of casein kinase 2 alpha (CSNK2A1) emerged as a potential upstream regulator of microtubule protrusions and a general regulator in the CDT response. Furthermore, we could prove for the first time CDT-mediated actin ribosylation on Arg-177.

## Materials and Methods

### Cell Culture

From HeLa cells, derived cell line HEp-2 was maintained in a 75 cm^2^ flask in a humidified atmosphere at 37°C and 5% CO_2_. Cells were cultured in minimal essential medium supplemented with 10% fetal bovine serum, 100 U/mL penicillin, and 100 U/mL streptomycin. Depending on confluency, the cells were split to maintain vitality.

### Toxin Treatment of HEp-2 Cells and Sample Preparation

Two days before toxin treatment, 7.5 × 10^5^ cells were seeded in 10 cm dishes to achieve a 75% confluency on the day of toxin treatment. CDTa and CDTb were generated using an *Escherichia coli* expression system as previously described (Beer et al., 2018). Cells were treated with 1.5 μg/mL CDTa and 3 μg/mL CDTb. Changes in the morphology were documented by phase-contrast microscopy after 4 and 8 h, respectively. For controls, only medium was exchanged. While LPS contamination was not separately checked by an assay, classical LPS reaction pathways, e.g., ERK pathway, show no activation (Supplementary Tables 1, 2), and no inflammatory GO terms were enriched in GO analysis indicating that there is no major additional effect concerning LPS. After documentation, cells were washed twice with ice-cold PBS and lysed by scraping them into 600 μL lysis buffer [8 M Urea, 50 mM ammonium bicarbonate (pH 8.0), 1 mM sodium ortho-vanadate, complete EDTA-free protease inhibitor cocktail (Roche), and phosphoSTOP phosphatase inhibitor cocktail (Roche)]. Lysates were sonicated on ice two times for 5 s at 30% energy. Cell debris was removed by centrifugation for 15 min at 16,100 × *g* at 4°C. The supernatant was collected and used for further preparation.

### Protein Digestion

Protein concentrations were estimated utilizing BCA assay (Thermo). Proteins were reduced with 5 mM DTT at 37°C for 1 h and afterward alkylated with 10 mM iodoacetamide at RT for 30 min. Alkylation was quenched by adding DTT to a final concentration of 5 mM. For digestion, lysates were diluted 1:5 with 50 mM ABC buffer to lower the concentration of urea below 2 M; 1 mg protein per condition was digested at 37°C for 4 h using 1:100 w/w Lys-C (Wako) followed by overnight digestion with 1/100 w/w trypsin (Promega). Digestion was stopped by adding TFA to a final concentration of 1%. Peptide solutions were then desalted with Sep Pak C18 1cc cartridges (Waters).

### Phosphopeptide Enrichment and Tandem Mass Tags Labeling

Before phosphopeptide enrichment, 25 μg of peptide of each condition was set aside for the proteome measurement. Phosphopeptide enrichment was performed following Sequential Enrichment from Metal Oxide Affinity Chromatography protocol (Thermo Scientific). Phosphopeptides have to be enriched since they only resemble a small proportion of all peptides and could rarely be measured without prior enrichment. Afterward, enriched phosphopeptides were desalted by C18 spin tips. BCA assay was used before labeling to estimate the peptide concentration of each sample. Equal amounts of enriched phosphopeptides and peptides for the proteomic analysis were labeled and combined following manufactures instructions. TMT labeling is performed to quantify multiple samples in one LC-MS run and minimize technical variability between samples. After labeling, peptides were fractionated into eight fractions by high pH fractionation (Thermo Scientific). Subsequently, peptides were vacuum dried and stored at −80°C until LC-MS measurement.

### LC-MS Analysis

Samples were dissolved in 0.1% TFA/2% ACN and analyzed in an Orbitrap Fusion Lumos mass spectrometer (Thermo Fisher Scientific) equipped with a nanoelectrospray source and connected to an Ultimate 3000 RSLC nanoflow system (Thermo Fisher Scientific). Peptides were loaded on an Acclaim PepMap C18 trap (Thermo Fisher Scientific) and separated by a 50 cm μPAC^TM^ (PharmaFluidics) analytical column at 35°C column temperature. Utilizing 0.1% formic acid as solvent A and 100% ACN with 0.1% formic acid as solvent B, we used a 120-min gradient at a flow rate of 500 nl/min ramping from 3.4% B to 21% B in 65 min, to 42% B in 32 min and to 75.6% B in 2 min, kept for 3 min, then to 3.6% B in 2 min, held for 16 min. The spray voltage was set to 2 kV. A data-dependent acquisition method, in which ions are selected on the basis of their MS1 signal and isolated for further fragmentation, was used with a cycle time of 3 s and Top N setting. Dynamic exclusion was set to 60 s, AGC target at 4 × 10^5^, maximum injection time at 50 ms, and Orbitrap resolution at 120,000 for MS1 scan. For all runs, an MS2 method was used with higher-energy-collisional-dissociation (HCD) fragmentation at 38% [for specific remeasurement for ribosylation verification collision-induced dissociation (CID) at 35% was used], first mass at 100 m/z, MS^2^ maximum injection time of 110 ms, MS^2^ isolation width at 0.8 m/z, and an Orbitrap resolution of 60,000. HCD and CID are fragmentation techniques that are used to fragment ions in gas phase to assess their composition and consequently to identify them. While CID is considered to use low energy for fragmentation and hence produce less fragmented ions, HCD fragmentation generates heavier fragmented ions and is very well suited to identify phosphorylated peptides including annotation of the phosphorylation site (Jedrychowski et al., 2011; Tu et al., 2016). For an in-depth review of current mass spectrometry techniques, we refer to Sinha and Mann (2020).

### Data Processing

Raw data were processed with MaxQuant software (version 1.6.3.3; Cox and Mann, 2008) using the Andromeda search engine (Cox et al., 2011). Spectra were searched against the Swiss-Prot reviewed UniprotKB database (version 01/2021, 20,395 entries; The UniProt Consortium et al., 2021). Carbamidomethylation of cysteine was set as fixed modification, and as variable modifications, oxidation of methionine, N-terminal acetylation, deamidation of glutamine, and asparagine were set. To detect mono-ADP-ribosylation (541.0611 Da, H_21_O_12_C_15_N_5_P_2_), this variable modification was set at cysteine, aspartate, glutamate, histidine, arginine, lysine, serine, threonine, and tyrosine residues. Similarly, phosphorylation (PO_4_) was set at serine, threonine, and tyrosine residues as variable modification. False discovery rate was set to 0.01 and maximum of missed cleavage to 2. Only phosphosites and proteins were used for quantification that were measured in all three replicates. Measured phosphosites with a localization probability below 75% were excluded from further processing. Both proteome and phosphoproteome were normalized by subtracting the median intensity of each sample and the median intensity of each TMT-batch, respectively. Only phosphosites were later included in quantitative analysis that could be normalized using the corresponding protein. Data evaluation, analysis, and visualization were done using Perseus (version 1.6.2.3.; Tyanova et al., 2016). The upstream analysis was generated through ingenuity pathway analysis (IPA, QIAGEN Inc., https://www.qiagenbio-informatics.com/products/ingenuity-pathway-analysis). The IPA software predicts regulatory proteins by comparing up- and down-regulated proteins and phosphorylation events to a hand-curated database and can therefore predict regulatory proteins that could be relevant to the observed proteomic and phosphoproteomic changes. All significant regulated sites were included. R Core Team (2013) in particular, the R packages complex heat map (Gu et al., 2016) and ggplot2 (Wickham, 2016) were used for data analysis and visualization. For heat map generation, protein and phosphosite intensities were used that were tested in a Benjamini Hochberg FDR-based ANOVA. For gene ontology analysis, the STRING data base (Jensen et al., 2009) was used. The mass spectrometry proteomics data have been deposited to the ProteomeXchange Consortium via the PRIDE (Griss et al., 2016) partner repository with the dataset identifier PXD027411.

## Results

### Alteration of Morphology After Treatment With CDT

**FIGURE 1:**
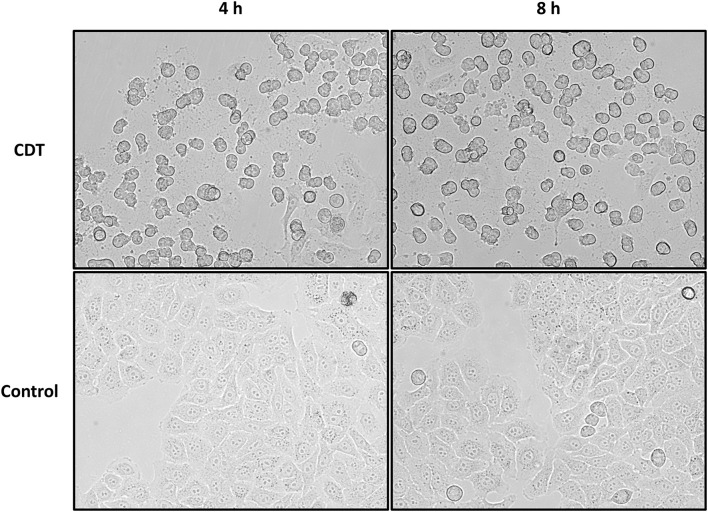
Morphology changes of HEp-2 cells after 4 and 8 h of CDT treatment. Cells were treated with 1.5 μg/mL of CDTa and 3 μg/mL of CDTb; controls were untreated.

Different concentrations of CDT for treatment of HEp-2 cells were tested, and 1.5 μg/mL was considered to be most suitable for the proteome and phosphoproteome experiments. After 4 h of treatment with CDT, most cells exhibit a rounded cell morphology but appear to be attached to the cell culture flask (Figure 1). After 8 h, rounding was more pronounced, and a small number of cells had detached from the ground. Detachment seemed to be a dynamic process that started only at some areas of cells, and many cells in the cell culture flasks were observed to be a little agile and seemed to stick only with a small part to the cell culture bottom while most of the cell was rounded and moved a little bit in the medium after easy panning (Figure 1).

### Quantitative Proteome Analysis After CDT Treatment

**FIGURE 2:**
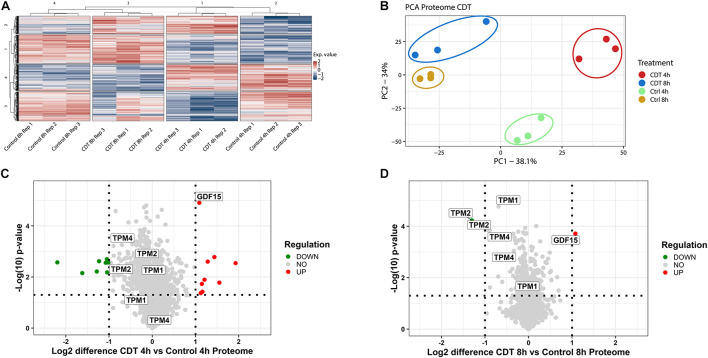
Proteome analysis of with CDT-treated HEp-2 cells after 4 and 8 h. **(A)** Heat map of proteins that were significantly changed (*p* < 0.05) after Benjamini Hochberg FDR-based ANOVA testing. **(B)** Principal component analysis (PCA) of proteins that were significantly altered (*p* < 0.05) after FDR-based ANOVA testing. Volcano plots of proteins with CDT treated Hep-2 cells after 4 h **(C)** and 8 h **(D)**, respectively. Proteins that have been labeled with the same name resemble the isoform of that protein **(C,D)**. [**(C,D)** Horizontal dotted line indicates a *p*-value threshold of *p* < 0.05, vertical line a twofold difference, green dots: twofold significant downregulation, red dots: twofold significant upregulation; **(A)** Exp. value: expression value; numbers of row and column cluster are for coordinative reasons and have no underlying meaning; **(B)** PC1: principal component 1; PC2: principal component 2].

**FIGURE 3:**
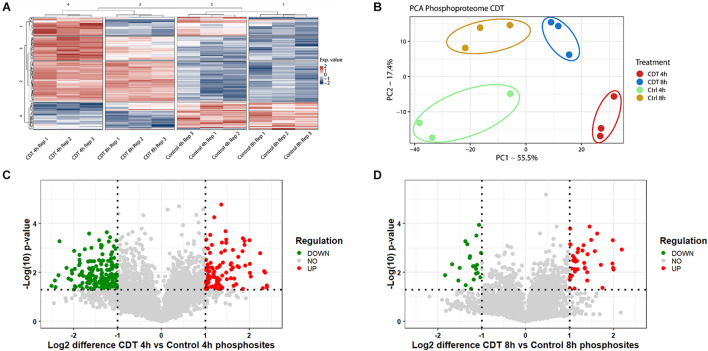
Phosphoproteome analysis of with CDT-treated HEp-2 cells after 4 and 8 h. **(A)** Heat map of significantly changed phosphosites (*p* < 0.05) based on intensity values and Benjamini Hochberg FDR ANOVA testing; Exp. value: expression value; numbers of row and column cluster are for coordinative reasons and have no underlying meaning. **(B)** Principal component analysis (PCA) of significantly changed phosphosites (*p* < 0.05) after FDR-based ANOVA testing (PC1: principal component 1; PC2: principal component 2). Volcano plots proteins of with CDT-treated HEp-2 cells after 4 h **(C)** and 8 h **(D)**, respectively, [**(C,D)** Horizontal dotted line indicates *p*-value threshold of *p* < 0.05, vertical line twofold difference, green dots: twofold significant downregulation, red dots: twofold significant upregulation].

The proteome was analyzed in a 4-plex TMT-based approach. In all three replicates, 4,348 proteins could be identified and quantified and were included in bioinformatic analysis. To assess evaluability, the data were collapsed into only two components [e.g., principal component 1 (PC1) and principal component 2 (PC2)], which explains most of their changes; the percentage sign after each component represents the percentage of the underlying data that this component describes (see Figures 2B, 3B). The different time points and treatments are then compared to each other to determine whether they can be differentiated. All different time points and conditions were distinguishable via PCA and, therefore, feasible for further analysis (Figure 2). Only minor changes could be detected in the proteome after 4 and 8 h (Figure 2), respectively. Several proteins were significantly altered but 99% with a ratio of less than twofold. Notably, after 8 h, tropomyosin subunits (TPM1, TPM2, and TPM4) were significantly down-regulated by 40% compared to the untreated 8-h sample and also down-regulated compared to the 4-h timepoint (Figure 2).

### Quantitative Phosphoproteome Analysis After CDT Treatment

In all three replicates, 5,686 phosphosites could be measured and 5,516 with a localization probability above 75%. Only phosphosites were included in a quantification that could be normalized by a corresponding protein to prohibit false hits that were only based on changed abundance of a protein; 4,619 phosphosites could be taken into analysis.

**FIGURE 4:**
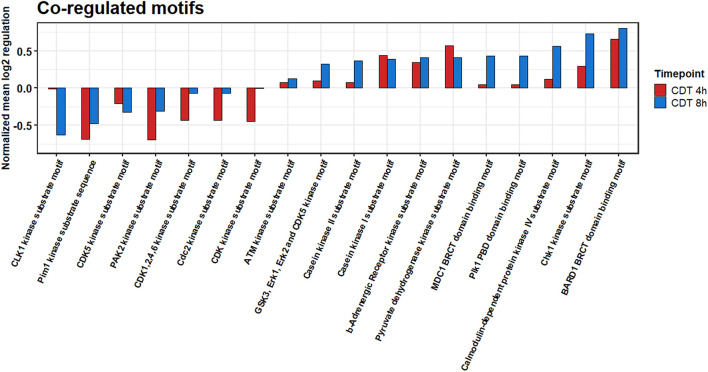
Summed co-regulated phosphorylation of motifs of significantly changed phosphosites after 4 and 8 h of CDT treatment.

All different time points and conditions were distinguishable *via* PCA and, therefore, feasible for further analysis (Figure 3). 330 phosphosites were significantly regulated after 4-h treatment compared to control and, after 8 h, 61 phosphosites with a regulation above twofold change, respectively. 200 (54 with a twofold change) phosphosites significantly changed at both time points compared to the control. The top 20 significant regulated phosphosites of each condition are shown in Supplementary Tables 3, 4. A Fisher’s exact test of the ANOVA significant phosphosites was performed to get an overview of the underlying kinase activation status. Enriched motifs were then analyzed on their phosphorylation status across the significantly changed phosphosites of both time points (Supplementary Figure 1). Motif phosphorylation that was regulated in the same direction at both time points was considered to play a role as a primary mechanism regulating the cell response to CDT and was therefore called “co-regulated.” Down-regulated phosphorylation was primarily observed on CDK and PIM1 based motifs in both time points. Overall, up-regulated phosphorylation was detected for Casein kinase family motifs as well as BARD1 BRCT motifs (Figure 4).

### Analysis of CSNK2A1 as a Potential Upstream Regulator

**FIGURE 5:**
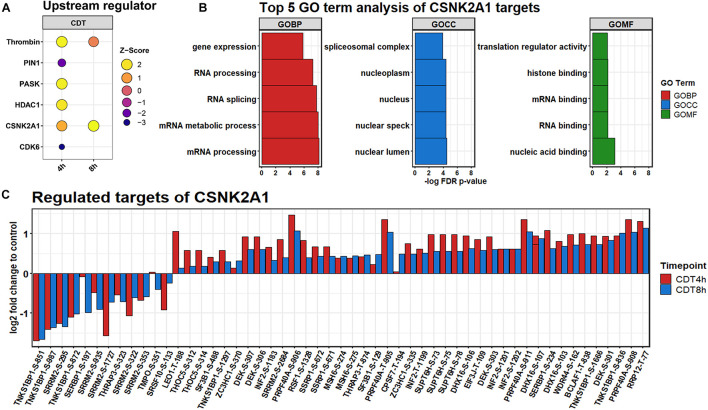
**(A)** Upstream regulators predicted by IPA^TM^. **(B)** Regulation of significantly changed predicted downstream phosphosite targets of CSNK2A1 after 4 h (red) and 8 h (blue) of CDT treatment. **(C)** GO analysis of predicted targets.

All significantly changed phosphosites of both time points were analyzed via the IPA^TM^ software. Based on a hand-curated database, IPA^TM^ predicts upstream regulator for phosphosites. A *Z*-score of above 2 or below −2 is considered activated or inhibited. For the 4 h time point; PIN1 and CDK6 were identified to be down-regulated. Thrombin, PASK, and HDAC1 were considered to be strongly up-regulated, and CSNK2A1 was slightly activated (Figure 5). For the 8 h time point, only CSNK2A1 was considered to be activated, and Thrombin was somewhat up-regulated. Since CSNK2A1 was regulated at both time points and phosphorylation of Casein kinase motifs was enhanced at both time points, potential downstream targets were further analyzed. IPA^TM^ predicts 28 proteins to be controlled by CSNK2A1. Significantly changed phosphosites containing the casein kinase II motif are targets of CK2 according to the PhosphoSitePlus^®^ database (Hornbeck et al., 2015), and were analyzed on their gene ontology using the STRING database (Jensen et al., 2009). Highly enriched were GO-terms that were part of the translational process like “RNA splicing” and “translational regulator activity.” The 30 most enriched GO terms for each time point are depicted in Supplementary Figures 3, 4.

### Detection of Actin Ribosylation on Arg-177

**FIGURE 6:**
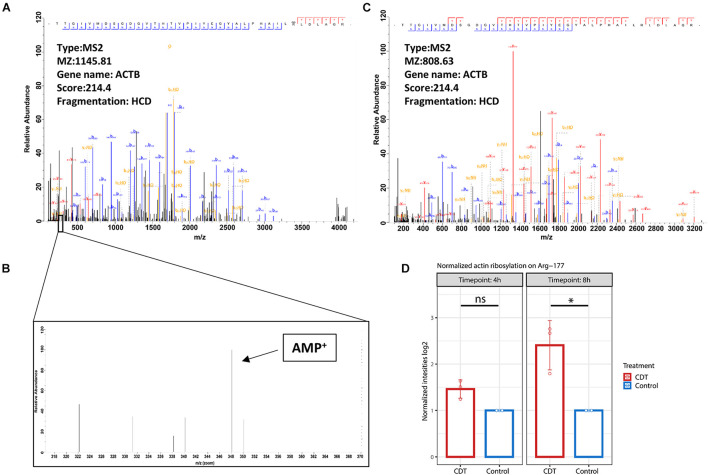
**(A)** Fragment spectrum of ADP ribosylated actin on Arg-177 on the tryptic peptide TTGIVMDSGDGVTHTVPIYEGYALPHAILRLDLAGR, **(C)** the unmodified version, and **(B)** diagnostic peak of AMP at 348.2 m/z. **(D)** Normalized intensity of ADP ribosylation on Arg-177 after 4- and 8-h treatment with CDT (*p < 0.05, ns: non-significant).

The mono-ADP-ribosylation could be detected at Arg-177 proved by an MS2 spectrum with an Andromeda score of 214.4. Interestingly, no fragment ion could be seen on Arg-177 at HCD 38% despite the 348.02 peak of AMP (Figure 6). At CID 35%, an overlapping ion could confirm the ribosylation at Arg-177 (Figure 6). Furthermore, the ribosylation could be quantified with an insignificant 1.4-fold increase after 4 h of treatment and a significant 2.8-fold increase after 8 h compared to control time points (Figure 6).

## Discussion

For the first time, the effects of CDT on the proteome and phosphoproteome of target cells have been examined.

Although the proteome remains only slightly changed, distinct proteins involved in modeling the cytoskeleton were regulated in a time-dependent manner between 4 and 8 h. Tropomyosin TPM1, TPM2, TPM4, and their isoforms were significantly down-regulated during 8 h of toxin treatment and compared to the 4 h time point with a clear downward shift (Figure 2). Tropomyosins have been shown to be essential regulators of the actin filament function (Gunning et al., 2015). In knockout cells, it has been demonstrated that CSNK2A1 controls the abundance of tropomyosin negatively (D’Amore et al., 2019). Furthermore, the cytokine GDF-15 that promotes cell migration is significantly twofold up-regulated at both time points (Figure 2), and its expression is indirectly controlled by CK2 (Feng et al., 2013).

The phosphoproteome was drastically altered in contrast to the proteome. Only enriched kinase motifs were analyzed with the same phosphorylation pattern at both time points to get an overview of the underlying kinase activation status over the given period. Cyclin-dependent kinases and Pim1 motifs were less phosphorylated (Figure 4), and CDK6 and PIN1 also appeared as down-regulated in their activity, as evident from the IPA^TM^ analysis (Figure 5). This downregulation was expected since it has been described for TcdA and TcdB that both can cause cell cycle arrest due to the collapse of the cytoskeleton that is also facilitated by CDT (Chandrasekaran and Lacy, 2017). Also, the beta-adrenergic receptor motif was stronger when phosphorylated together with the BARD1 motif. These regulations could also be foreseen considering that CDT has been described to activate the NFκB-pathway and the induction of apoptosis (Chandrasekaran and Lacy, 2017; Simpson et al., 2020).

Noticeably, changes of the phosphoproteome at the 4 h time point are more drastic than at the 8 h time point. This observation was apparently due to the collapse of the cytoskeleton between 3 and 4 h after treatment (data not shown). Accordingly, the time point of measurement was set to 4 h to see the detailed cell reaction. At the 4 h time point, many processes were the target of imminent downregulation (e.g., cell cycle) and upregulation of damage response regulators, e.g., HDAC1 (Figure 5A; Miller et al., 2010). Consequently, the summed phosphorylation of different motifs compared to the control for the 4 h time point were primarily negative and do not correlate with the 8 h time point (Supplementary Figure 1). At the 8 h time point, those sudden changes were no longer present, and the underlying mechanism of the cell response to CDT could be viewed as more pronounced. So, only the phosphorylation of motifs regulated in the same direction was considered to describe this hidden mechanism and was further analyzed.

The phosphorylation of casein kinase motifs was up-regulated at both time points, and CSNK2A1 was strongly induced as an upstream regulator (Figure 5). According to a gene ontology analysis, CSNK2A1 targets that were analyzed in this dataset were mainly involved in transcriptional processes (Figure 5). Together with the regulated proteins, this implied an activated CSNK2A1 kinase.

A recent study suggests that SEPTINs were drivers of microtubule-based protrusions (Nölke et al., 2016). Although we did only check on general morphological changes as a marker of the CDT effect and did not look specifically for protrusions, it is noteworthy that SEPT2 is a target of CSNK2A1 with a phosphorylation site at S228. This site was found significantly altered in ANOVA-FDR-based testing and slightly up-regulated but not significant at both time points (Supplementary Table 3). This phosphorylation has been shown to positively influence the GTP binding of SEPTINs (Huang et al., 2006). Additionally, it has been demonstrated that in CSNK2A1 knockout cell, SEPTIN2 is significantly down-regulated (Borgo et al., 2017). This indicates a distinct regulatory mechanism, via CSNK2A1, SEPTIN, and GTPases, that could steer the microtubule polymerization and cause protrusions in the CDT setting.

While general actin ribosylation by CDT has already been shown (Gülke et al., 2001), here we could confirm for the first time that CDT ribosylates actin specifically on Arg-177. Interestingly, HCD fragmentation produces no fragment ion containing the ribosylation site on Arg-177, despite detecting the mono-ADP-ribose fragment at 348.2 m/z (Figure 6), described as a diagnostic ADP ribosylation marker (Schröder et al., 2018). However, the CID fragmentation method produced fragment ions containing the modification and served as an additional verification on the localization of the ADP ribosylation (Supplementary Figure 2).

This study offers a first glimpse of the underlying mechanisms that concern the detailed cell response to CDT. However, more experiments must be made to solidify those first suggestions that CSNK2A1 acts as an upstream regulator by, e.g., knockout experiments following experiments with primary cells instead of cell lines. In further investigations, it would also be beneficial to add more time points, especially to identify phosphorylation changes very early, e.g., 10–60 min after toxin addition. Of exceptional interest in those early time points would be the lipolysis-stimulated lipoprotein receptor (LSR) described hitherto as the only receptor for CDT (Papatheodorou et al., 2011) which could also act as an early upstream regulator. We found the phosphorylation of LSR on site S643 with a CSNK2A1 motif significantly altered after 4 h (data not shown), but since have not measured the corresponding protein, we could not include this into our analysis. Yet, it could hint that CSNK2A1 and LSR are connected and interact in a feedback loop. A different methodical setup must be applied in the following study to take receptor phosphorylation status into account since membrane proteins are challenging to analyze *via* LC-MS-based proteomics (Vit and Petrak, 2017).

Taken together, we describe the first phosphoproteome and proteome analysis after treatment of cells with CDT and offer first insights into the cellular response to this toxin. More studies have to be made to deepen these findings and elaborate on them further.

## Data Availability Statement

The dataset presented in this study has been deposited to the ProteomeXchange Consortium via the PRIDE (Griss et al., 2016) partner repository with the dataset identifier PXD027411.

## Author Contributions

AP designed and coached the experiments, analyzed the data, and wrote and submitted the manuscript. FS designed and performed the experiments, analyzed the data, and wrote the manuscript. RG produced the toxins and supported the experiments. All authors contributed to the article and approved the submitted version.

## Conflict of Interest

The authors declare that the research was conducted in the absence of any commercial or financial relationships that could be construed as a potential conflict of interest.

## Publisher’s Note

All claims expressed in this article are solely those of the authors and do not necessarily represent those of their affiliated organizations, or those of the publisher, the editors and the reviewers. Any product that may be evaluated in this article, or claim that may be made by its manufacturer, is not guaranteed or endorsed by the publisher.

## Abbreviations

DDA: Data-dependent acquisition
CID: Collision-induced dissociation
HCD: Higher-energy-collisional-dissociation
TMT: Tandem mass tags
PCA: Principal component analysis
IPA ^TM^: Ingenuity Pathway Analysis ^TM^
ACN: Acetonitrile
AGC: Automatic gain control.
