# Supplementary material for: The Binary Toxin of Clostridioides difficile Alters the Proteome and Phosphoproteome of HEp-2 Cells
Source: Front Microbiol. 2021 Sep 14;12:725612. doi: 10.3389/fmicb.2021.725612 (PMC8477661; doi:10.3389/fmicb.2021.725612)
Supplement: Supplementary file 1 [file Data_Sheet_1.docx]

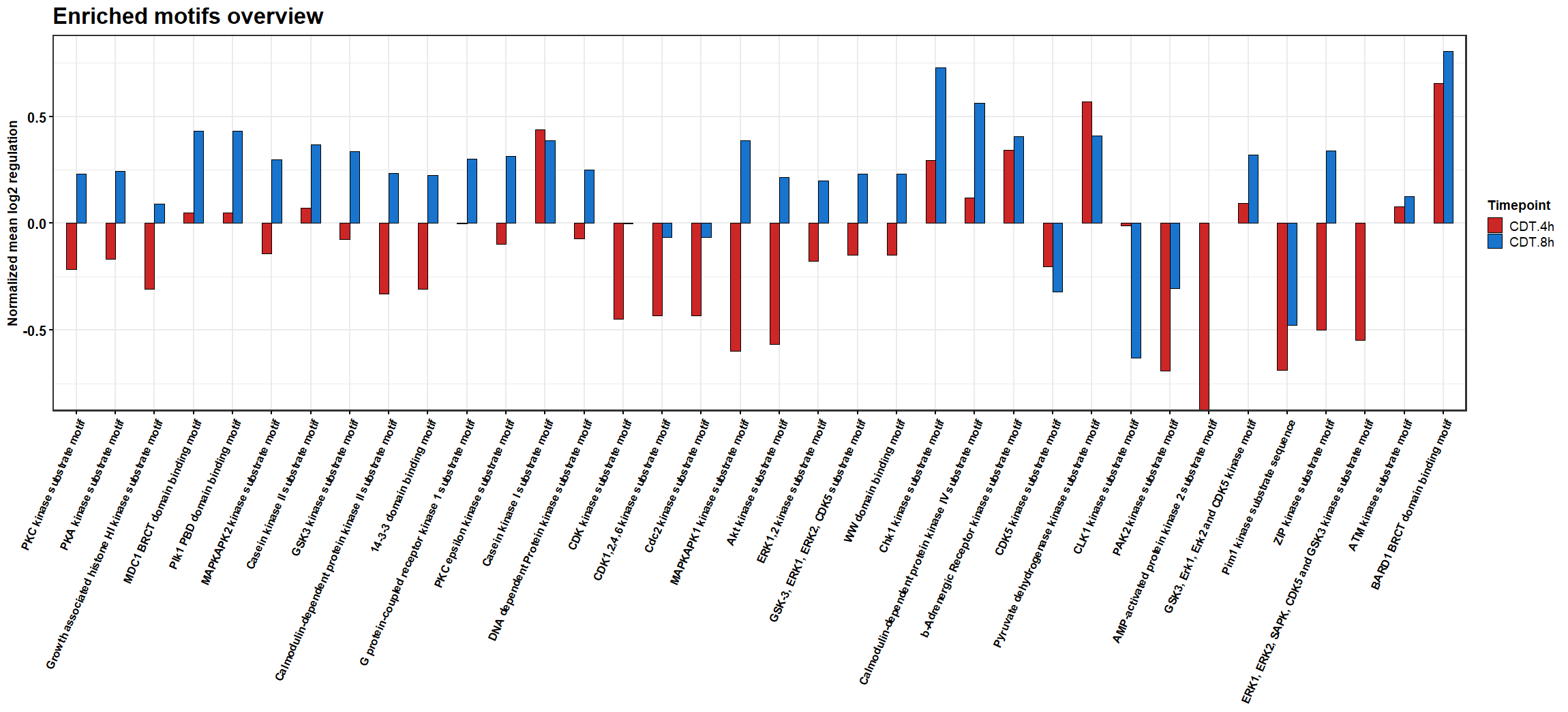


**Supplementary figure 1:** Summed regulation of regulated motifs of significantly changed phosphosites after 4 h and 8 h of CDT treatment.
