# Supplementary material for: The Binary Toxin of Clostridioides difficile Alters the Proteome and Phosphoproteome of HEp-2 Cells
Source: Front Microbiol. 2021 Sep 14;12:725612. doi: 10.3389/fmicb.2021.725612 (PMC8477661; doi:10.3389/fmicb.2021.725612)
Supplement: Supplementary file 2 [file Data_Sheet_2.docx]

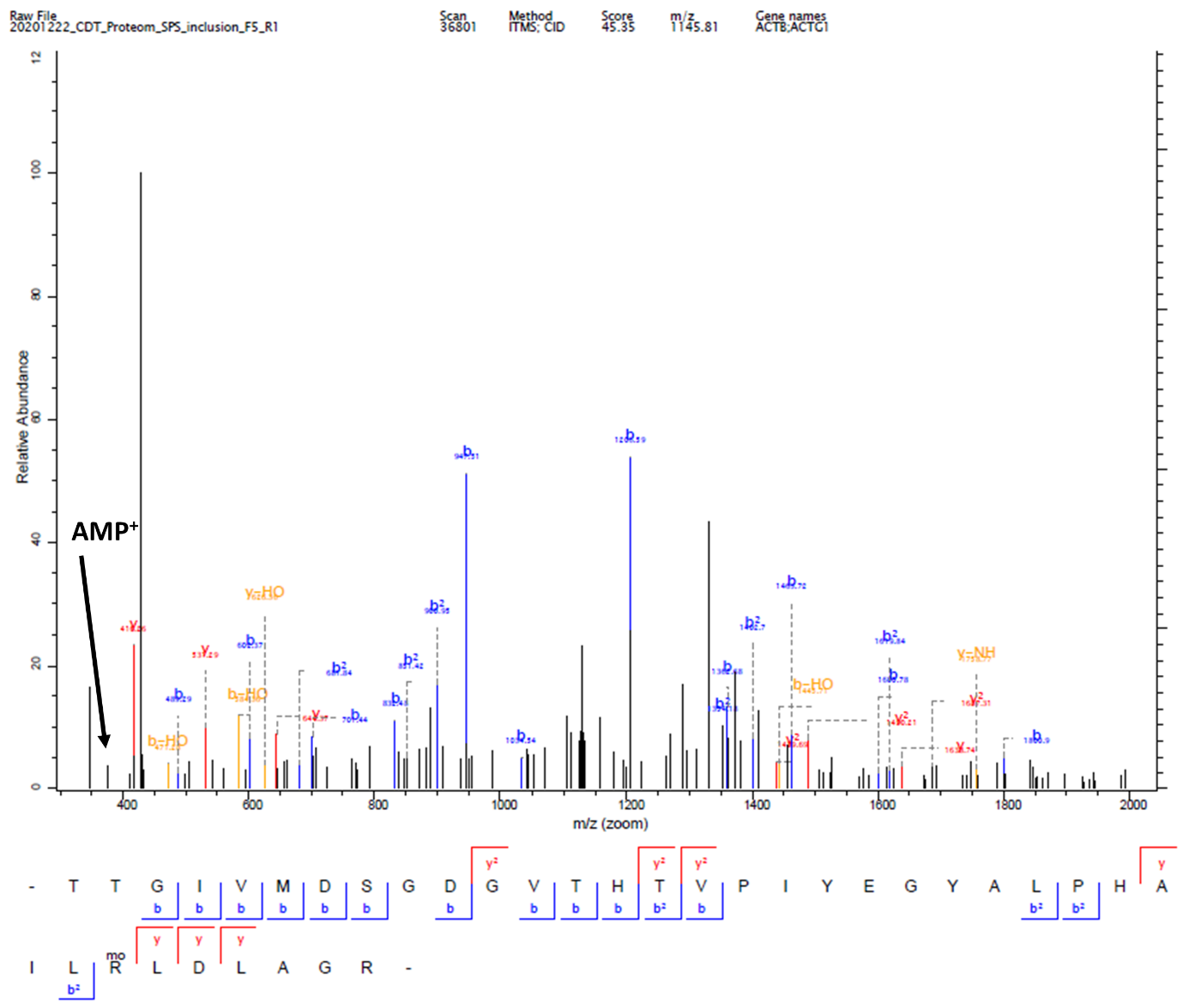


**Supplementary figure 2:** Fragment spectrum of ADP ribosylated actin on Arg-177 on the tryptic peptide TTGIVMDSGDGVTHTVPIYEGYALPHAILRLDLAGR with CDI at 35% and diagnostic peak of AMP at 348.2.
