# Supplementary material for: The Binary Toxin of Clostridioides difficile Alters the Proteome and Phosphoproteome of HEp-2 Cells
Source: Front Microbiol. 2021 Sep 14;12:725612. doi: 10.3389/fmicb.2021.725612 (PMC8477661; doi:10.3389/fmicb.2021.725612)
Supplement: Supplementary file 4 [file Data_Sheet_4.docx]

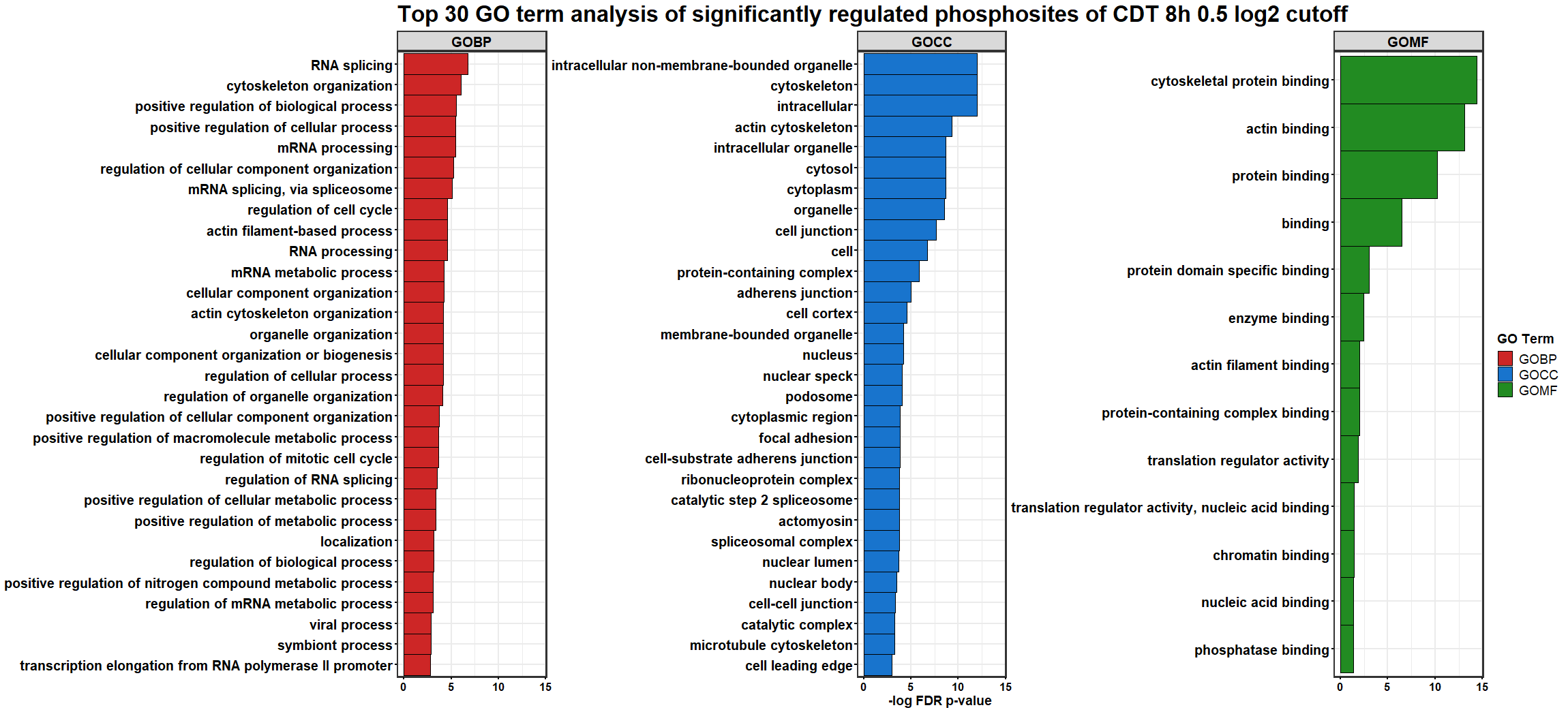


**Supplementary figure 4:** GO term analysis of significantly regulated phosphosites after 8 h of CDT treatment with a minimum change of 0.5 log2 change compared to control
