# Supplementary material for: The Binary Toxin of Clostridioides difficile Alters the Proteome and Phosphoproteome of HEp-2 Cells
Source: Front Microbiol. 2021 Sep 14;12:725612. doi: 10.3389/fmicb.2021.725612 (PMC8477661; doi:10.3389/fmicb.2021.725612)
Supplement: Supplementary file 5 [file Table_1.docx]

**Supplementary Table 1:** Phosphosites and porteins that are prominent inflammatory marker for LPS reaction

| Gene name | P-Site | p-value 8h CDT vs 8h Ctrl | log2 difference 8h CDT vs 8h Ctrl | p-value 4h CDT vs 4h Ctrl | log2 difference 4h CDT vs 4h Ctrl |
| --- | --- | --- | --- | --- | --- |
| MAP2K1 | S298 | 0.008871152 | -1.01852 | 0.01251901 | -1.63679 |
| STAT1 | S727 | 0.441696543 | 0.282629 | 0.838825453 | -0.0700467 |
| STAT3 | S727 | 0.077735608 | 0.487965 | 0.063238273 | 0.352707 |
| IL-18 | NA | 0.930193499 | 0.00161489 | 0.006470383 | -0.0499064 |
