# Supplementary material for: The Binary Toxin of Clostridioides difficile Alters the Proteome and Phosphoproteome of HEp-2 Cells
Source: Front Microbiol. 2021 Sep 14;12:725612. doi: 10.3389/fmicb.2021.725612 (PMC8477661; doi:10.3389/fmicb.2021.725612)
Supplement: Supplementary file 6 [file Table_2.docx]

**Supplementary table 2:** IPA upstream regulator analysis of proteins that are responsible for LPS response for 4h and 8h time point

| Gene Name | IPA activation Z-score 8h CDT vs 8h Ctrl | IPA activation Z-score 4h CDT vs 4h Ctrl |
| --- | --- | --- |
| MAP2K1 | -0.747 | -1.253 |
| ERK | -0.243 | -0.382 |
