# Supplementary material for: The Binary Toxin of Clostridioides difficile Alters the Proteome and Phosphoproteome of HEp-2 Cells
Source: Front Microbiol. 2021 Sep 14;12:725612. doi: 10.3389/fmicb.2021.725612 (PMC8477661; doi:10.3389/fmicb.2021.725612)
Supplement: Supplementary file 7 [file Table_3.docx]

**Supplementary table 3:** Top 20 up- and downregulated phosphosites after 4h CDT treatment

| Gene name | Protein name | *p*-value | Log_2_ ratio CDT 4 h/Control 4 h | Score | Amino acid |
| --- | --- | --- | --- | --- | --- |
| PTK2 | Focal adhesion kinase 1 | 0.0303576 | -2.88897069 | 73.502 | S-794 |
| ANP32B | Acidic leucine-rich nuclear phosphoprotein 32 family member B | 0.02047325 | -2.77569326 | 167.01 | T-244 |
| ZC3H18 | Zinc finger CCCH domain-containing protein 18 | 0.03404746 | -2.6094799 | 101.3 | T-851 |
| RBM15 | Putative RNA-binding protein 15 | 0.03812913 | -2.43663756 | 145.52 | S-612 |
| WASL | Neural Wiskott-Aldrich syndrome protein | 0.00048616 | -2.34405073 | 85.808 | S-430 |
| TRA2B | Transformer-2 protein homolog beta | 0.03442335 | -2.1924092 | 47.75 | S-16 |
| SRRM2 | Serine/arginine repetitive matrix protein 2 | 0.00708274 | -2.14314111 | 91.313 | S-1866 |
| PALLD | Palladin | 0.017728 | -2.11845907 | 118.28 | S-1104 |
| RBBP6 | E3 ubiquitin-protein ligase RBBP6 | 0.04504928 | -2.02702014 | 122.16 | S-839 |
| SRRM2 | Serine/arginine repetitive matrix protein 2 | 0.01174064 | -2.01456483 | 66.393 | S-1854 |
| PRPF4B | Serine/threonine-protein kinase PRP4 homolog | 0.04882896 | -2.00354067 | 109.16 | S-368 |
| SRSF4 | Serine/arginine-rich splicing factor 4 | 0.02107826 | -1.97171879 | 82.261 | S-330 |
| SRSF4 | Serine/arginine-rich splicing factor 4 | 0.02107826 | -1.97171879 | 82.261 | S-332 |
| SRRM2 | Serine/arginine repetitive matrix protein 2 | 0.00129193 | -1.96639347 | 88.448 | S-1822 |
| SRRM2 | Serine/arginine repetitive matrix protein 2 | 0.04079194 | -1.95306142 | 59.35 | S-1764 |
| SRRM1 | Serine/arginine repetitive matrix protein 1 | 0.00339405 | -1.94526704 | 93.345 | S-583 |
| SRRM1 | Serine/arginine repetitive matrix protein 1 | 0.00339405 | -1.94526704 | 93.345 | T-581 |
| CAP1 | Adenylyl cyclase-associated protein 1 | 0.0197006 | -1.9306186 | 133.32 | S-34 |
| SORBS3 | Vinexin | 0.00336335 | -1.9256827 | 137.85 | S-203 |
| EPS8L2 | Epidermal growth factor receptor kinase substrate 8-like protein 2 | 0.00390557 | -1.89239057 | 85.676 | T-184 |
| CD97 | CD97 antigen;CD97 antigen subunit alpha | 0.00554211 | 1.82540512 | 112.71 | S-738 |
| BAG3 | BAG family molecular chaperone regulator 3 | 0.01652943 | 1.886151 | 178.79 | S-136 |
| TLN2 | Talin-2 | 0.00131195 | 1.9009552 | 110.66 | T-1843 |
| WDR44 | WD repeat-containing protein 44 | 0.00082127 | 1.91099167 | 223.51 | T-271 |
| SUN2 | SUN domain-containing protein 2 | 0.01993567 | 1.95320574 | 103.69 | S-38 |
| LMO7 | LIM domain only protein 7 | 0.00069027 | 2.00466792 | 179.72 | T-598 |
| CTTN | Src substrate cortactin | 0.00796308 | 2.015426 | 259.07 | S-418 |
| SSR3 | Translocon-associated protein subunit gamma | 0.00028575 | 2.03049914 | 102.87 | S-105 |
| G3BP1 | Ras GTPase-activating protein-binding protein 1 | 0.01079039 | 2.05316099 | 424.07 | S-232 |
| AHNAK | Neuroblast differentiation-associated protein AHNAK | 0.00202264 | 2.23667463 | 289.45 | S-135 |
| HUWE1 | E3 ubiquitin-protein ligase HUWE1 | 0.01004335 | 2.4034907 | 278.07 | S-1898 |
| KNOP1 | Lysine-rich nucleolar protein 1 | 0.00705982 | 2.40536912 | 215.53 | T-47 |
| CD97 | CD97 antigen;CD97 antigen subunit alpha;CD97 antigen subunit beta | 0.00050759 | 2.42214076 | 139.38 | S-740 |
| HNRNPD | Heterogeneous nuclear ribonucleoprotein D0 | 0.03609494 | 2.45783043 | 362.39 | S-83 |
| GLYR1 | Putative oxidoreductase GLYR1 | 0.03159559 | 2.51179536 | 157.14 | S-114 |
| DSP | Desmoplakin | 0.00111708 | 2.52031549 | 395.37 | S-2606 |
| NUCKS1 | Nuclear ubiquitous casein and cyclin-dependent kinase substrate 1 | 0.02374049 | 2.65050507 | 112.45 | S-40 |
| TLE3 | Transducin-like enhancer protein 3 | 0.00858889 | 2.69041475 | 196.55 | T-321 |
| MDC1 | Mediator of DNA damage checkpoint protein 1 | 0.02378277 | 2.80102762 | 259.74 | S-453 |
| MDC1 | Mediator of DNA damage checkpoint protein 1 | 0.02378277 | 2.80102762 | 259.74 | T-455 |
