# Supplementary material for: The Binary Toxin of Clostridioides difficile Alters the Proteome and Phosphoproteome of HEp-2 Cells
Source: Front Microbiol. 2021 Sep 14;12:725612. doi: 10.3389/fmicb.2021.725612 (PMC8477661; doi:10.3389/fmicb.2021.725612)
Supplement: Supplementary file 8 [file Table_4.docx]

**Supplementary Table 4:** Top 20 up- and downregulated phosphosites after 8h CDT treatment

| Gene names | Protein names | p-value | Log2 ratio CDT 8 h/Control 8 h | Score | Amino acid |
| --- | --- | --- | --- | --- | --- |
| WASL | Neural Wiskott-Aldrich syndrome protein | 0.01634491 | -1.82486089 | 85.808 | S-430 |
| TNKS1BP1 | 182 kDa tankyrase-1-binding protein | 0.00635741 | -1.66352558 | 200.98 | S-851 |
| RRAGC | Ras-related GTP-binding protein C | 0.02399833 | -1.52716605 | 138.25 | T-394 |
| TNKS1BP1 | 182 kDa tankyrase-1-binding protein | 0.00091231 | -1.36056042 | 294.44 | S-987 |
| SRRM2 | Serine/arginine repetitive matrix protein 2 | 0.03621763 | -1.33595912 | 144.82 | S-295 |
| ARFGEF2 | Brefeldin A-inhibited guanine nucleotide-exchange protein 2 | 0.00125803 | -1.33157063 | 179.95 | S-1511 |
| STK10 | Serine/threonine-protein kinase 10 | 0.00137294 | -1.32521979 | 200.63 | S-454 |
| LIMA1 | LIM domain and actin-binding protein 1 | 0.01327943 | -1.31417561 | 281.12 | S-228 |
| EPS8L2 | Epidermal growth factor receptor kinase substrate 8-like protein 2 | 0.00374323 | -1.20715586 | 85.676 | T-184 |
| RANBP1 | Ran-specific GTPase-activating protein | 0.00505473 | -1.14460023 | 213.26 | T-15 |
| RANBP1 | Ran-specific GTPase-activating protein | 0.00350555 | -1.1430734 | 243.63 | T-13 |
| EGLN1 | Egl nine homolog 1 | 0.03251706 | -1.12974358 | 141.31 | S-125 |
| RANBP1 | Ran-specific GTPase-activating protein | 0.00554889 | -1.10673396 | 243.63 | S-14 |
| DBN1 | Drebrin | 0.00011767 | -1.02583377 | 294.97 | S-337 |
| TNKS1BP1 | 182 kDa tankyrase-1-binding protein | 0.01796302 | -1.02418423 | 186.51 | S-872 |
| MAP2K1 | Dual specificity mitogen-activated protein kinase kinase 1 | 0.00887105 | -1.01852099 | 139.79 | S-298 |
| TNKS1BP1 | 182 kDa tankyrase-1-binding protein | 0.00412814 | -1.00617154 | 266.7 | S-893 |
| SYNPO | Synaptopodin | 0.00795231 | -0.99850019 | 231.49 | T-856 |
| SERBP1 | Plasminogen activator inhibitor 1 RNA-binding protein | 0.04331465 | -0.98044364 | 162.69 | S-197 |
| YY1 | Transcriptional repressor protein YY1 | 0.03631696 | -0.97511673 | 80.755 | T-348 |
| HNRNPK | Heterogeneous nuclear ribonucleoprotein K | 0.00204433 | 1.24072679 | 300.57 | S-116 |
| ABLIM1 | Actin-binding LIM protein 1 | 0.00611824 | 1.26877371 | 242.07 | S-136 |
| EPS15 | Epidermal growth factor receptor substrate 15 | 0.00098026 | 1.30695629 | 102.01 | S-796 |
| USP5 | Ubiquitin carboxyl-terminal hydrolase 5 | 0.00347919 | 1.33749517 | 103.43 | T-623 |
| SSR3 | Translocon-associated protein subunit gamma | 0.02423039 | 1.34825802 | 102.87 | S-105 |
| EEF2 | Elongation factor 2 | 0.00574503 | 1.366841 | 161.19 | T-57 |
| MARCKS | Myristoylated alanine-rich C-kinase substrate | 0.00062918 | 1.382164 | 211.15 | S-118 |
| DSP | Desmoplakin | 0.00190297 | 1.40658188 | 395.37 | S-2606 |
| GORASP2 | Golgi reassembly-stacking protein 2 | 0.00399986 | 1.44163259 | 237.01 | T-433 |
| SPAG9 | C-Jun-amino-terminal kinase-interacting protein 4 | 6.7783E-05 | 1.48372777 | 201.66 | T-226 |
| WDR44 | WD repeat-containing protein 44 | 0.00781964 | 1.51640288 | 223.51 | T-271 |
| CD97 | CD97 antigen;CD97 antigen subunit alpha;CD97 antigen subunit beta | 0.00018737 | 1.56349818 | 112.71 | S-738 |
| LMO7 | LIM domain only protein 7 | 0.00130619 | 1.67243958 | 179.72 | T-598 |
| ABLIM1 | Actin-binding LIM protein 1 | 0.0018723 | 1.80152988 | 99.257 | S-304 |
| LMO7 | LIM domain only protein 7 | 0.03605668 | 1.84202321 | 188.26 | S-1159 |
| JUN | Transcription factor AP-1;Transcription factor jun-D | 0.00603427 | 1.95983823 | 190.03 | S-100 |
| CFL1 | Cofilin-1 | 0.00977083 | 1.97441483 | 120.12 | S-3 |
| AHNAK | Neuroblast differentiation-associated protein AHNAK | 0.0004488 | 2.05653254 | 289.45 | S-135 |
| PEA15 | Astrocytic phosphoprotein PEA-15 | 0.00583023 | 2.12026532 | 210.57 | S-116 |
| CD97 | CD97 antigen;CD97 antigen subunit alpha;CD97 antigen subunit beta | 0.00134895 | 2.12511381 | 139.38 | S-740 |
