# Supplementary material for: The Binary Toxin of Clostridioides difficile Alters the Proteome and Phosphoproteome of HEp-2 Cells
Source: Front Microbiol. 2021 Sep 14;12:725612. doi: 10.3389/fmicb.2021.725612 (PMC8477661; doi:10.3389/fmicb.2021.725612)
Supplement: Supplementary file 9 [file Table_5.docx]

**Supplementary table5:** Regulation of SEPT2 on phosphosite S-218

| Gene names | Protein names | ANOVA *p*-value | p-value CDT 4 h/Control 4 h | Log2 ratio CDT 4 h/Control 4 h | p-value CDT 8 h/Control 8 h | Log2 ratio CDT 8 h/Control 8 h | Amino acid |
| --- | --- | --- | --- | --- | --- | --- | --- |
| SEPT2 | Septin-2 | 0.044720321 | 0.06920121 | 0.75571442 | 0.24324448 | 0.20159976 | S-218 |
